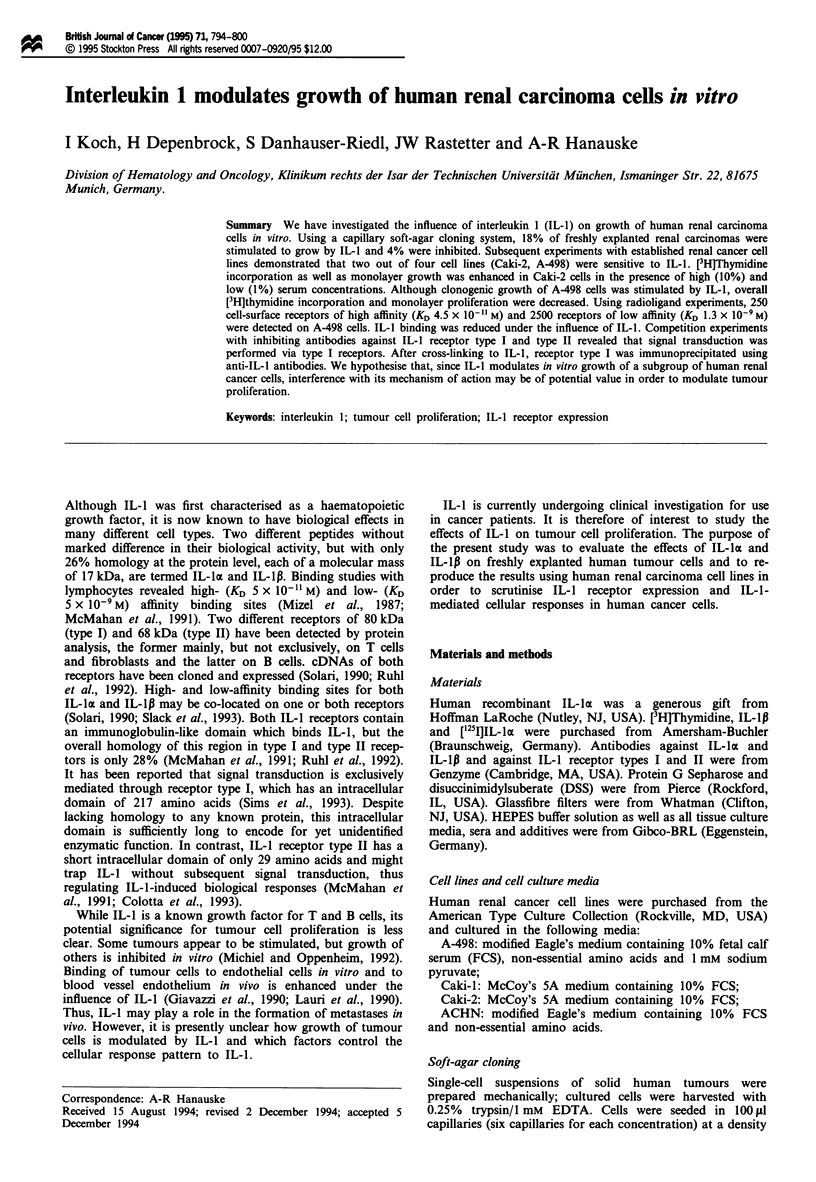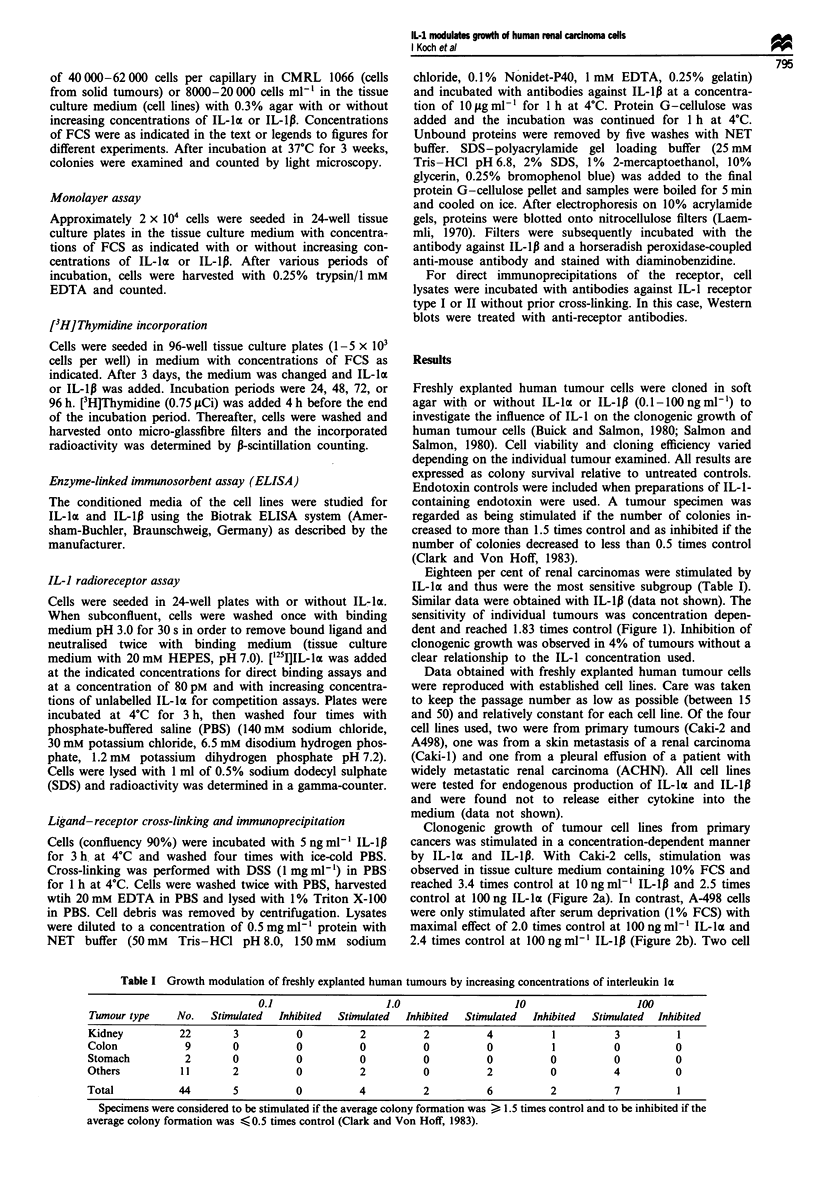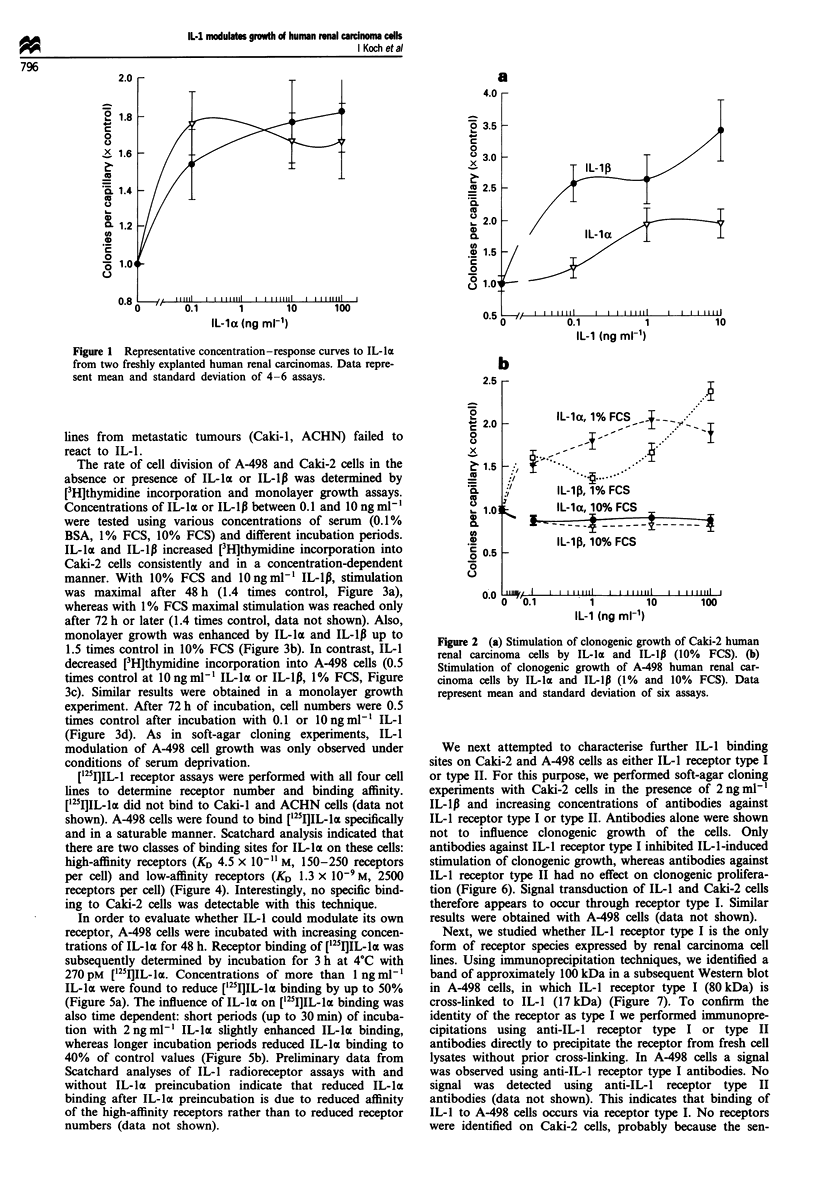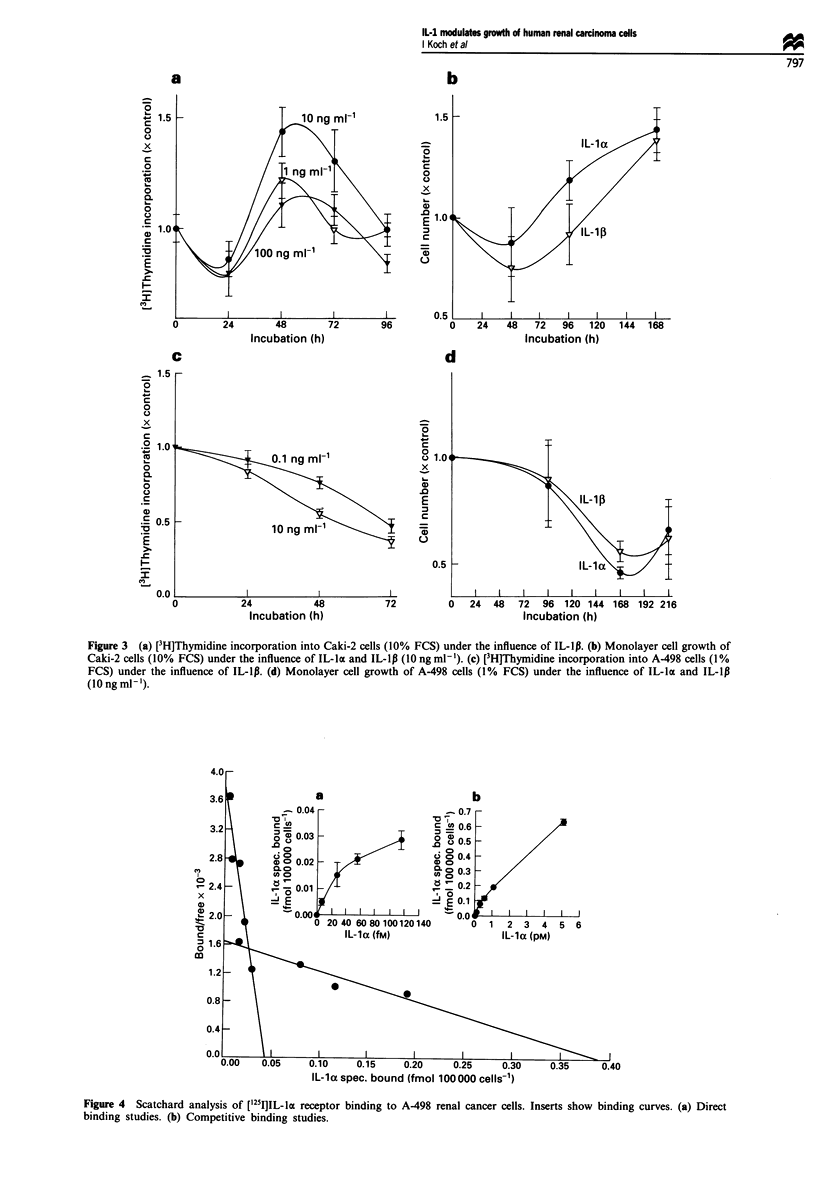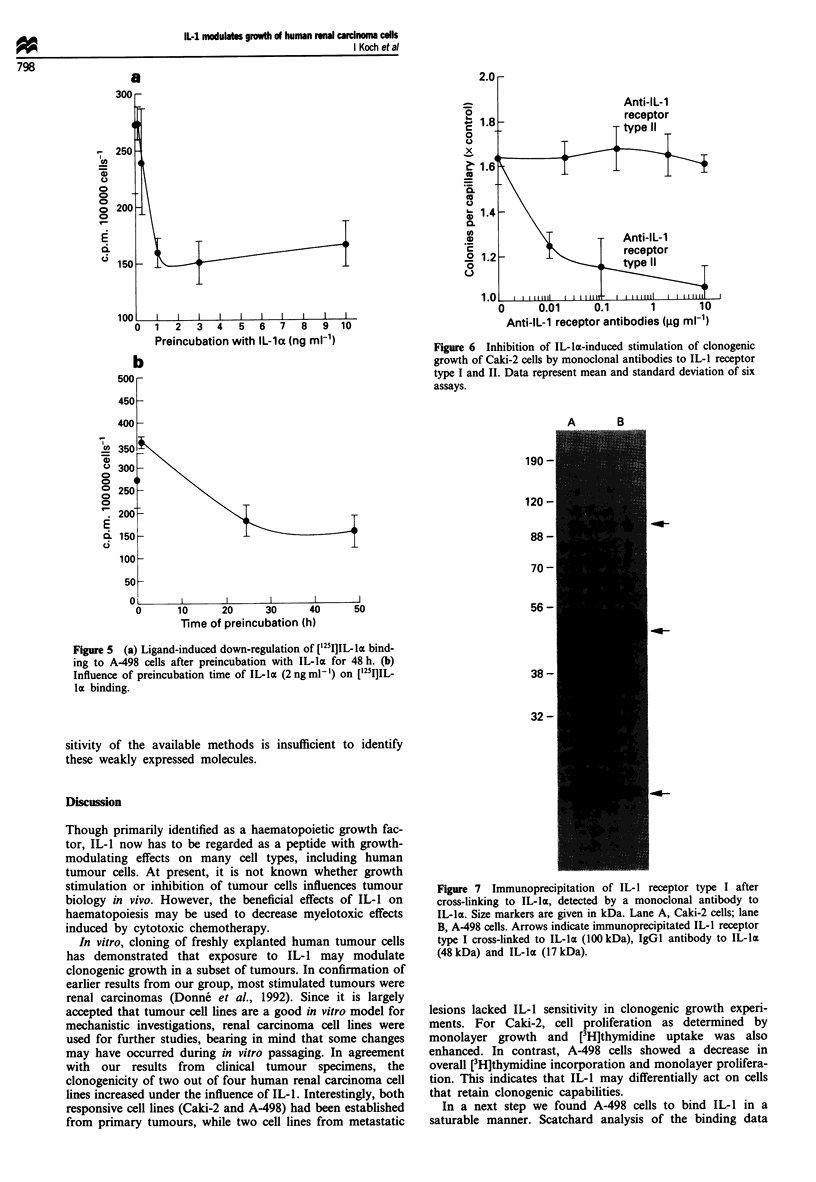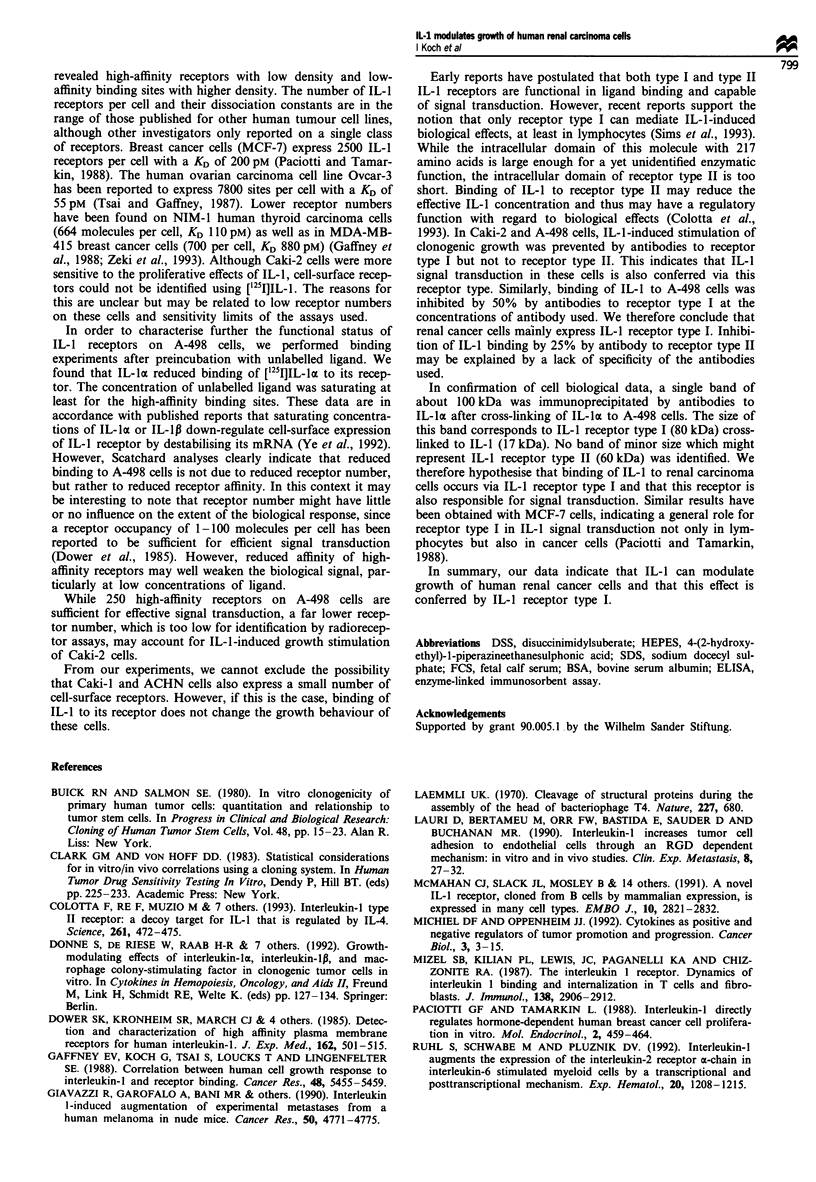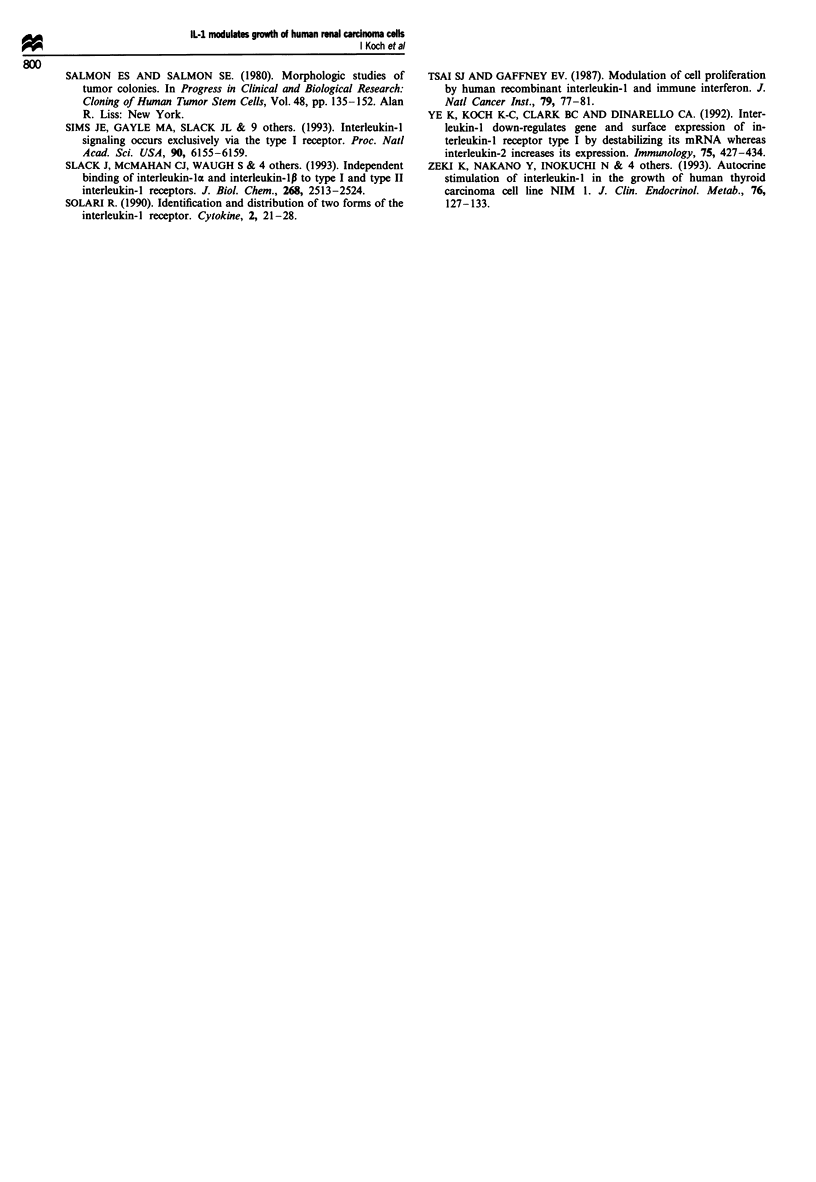# Interleukin 1 modulates growth of human renal carcinoma cells in vitro.

**DOI:** 10.1038/bjc.1995.154

**Published:** 1995-04

**Authors:** I. Koch, H. Depenbrock, S. Danhauser-Riedl, J. W. Rastetter, A. R. Hanauske

**Affiliations:** Division of Hematology and Oncology, Technischen Universität München, Germany.

## Abstract

**Images:**


British Journal of Cancer (1995) 71, 794-800

© 1995 Stockton Press All rights reserved 0007-0920/95 $12.00

### Interleukin 1 modulates growth of human renal carcinoma cells *in vitro*

I Koch, H Depenbrock, S Danhauser-Riedl, JW Rastetter and A-R Hanauske

*Division of Hematology and Oncology, Klinikum rechts der Isar der Technischen Universität München, Ismaninger Str. 22, 81675 Munich, Germany.*

**Summary** We have investigated the influence of interleukin 1 (IL-1) on growth of human renal carcinoma cells *in vitro*. Using a capillary soft-agar cloning system, 18% of freshly explanted renal carcinomas were stimulated to grow by IL-1 and 4% were inhibited. Subsequent experiments with established renal cancer cell lines demonstrated that two out of four cell lines (Caki-2, A-498) were sensitive to IL-1. [$^3$H]Thymidine incorporation as well as monolayer growth was enhanced in Caki-2 cells in the presence of high (10%) and low (1%) serum concentrations. Although clonogenic growth of A-498 cells was stimulated by IL-1, overall [$^3$H]thymidine incorporation and monolayer proliferation were decreased. Using radioligand experiments, 250 cell-surface receptors of high affinity ($K_D$ $4.5 \times 10^{-11}$ M) and 2500 receptors of low affinity ($K_D$ $1.3 \times 10^{-9}$ M) were detected on A-498 cells. IL-1 binding was reduced under the influence of IL-1. Competition experiments with inhibiting antibodies against IL-1 receptor type I and type II revealed that signal transduction was performed via type I receptors. After cross-linking to IL-1, receptor type I was immunoprecipitated using anti-IL-1 antibodies. We hypothesise that, since IL-1 modulates *in vitro* growth of a subgroup of human renal cancer cells, interference with its mechanism of action may be of potential value in order to modulate tumour proliferation.

**Keywords**: interleukin 1; tumour cell proliferation; IL-1 receptor expression

Although IL-1 was first characterised as a haematopoietic growth factor, it is now known to have biological effects in many different cell types. Two different peptides without marked difference in their biological activity, but with only 26% homology at the protein level, each of a molecular mass of 17 kDa, are termed IL-1α and IL-1β. Binding studies with lymphocytes revealed high- ($K_D$ $5 \times 10^{-11}$ M) and low- ($K_D$ $5 \times 10^{-9}$ M) affinity binding sites (Mizel *et al.*, 1987; McMahan *et al.*, 1991). Two different receptors of 80 kDa (type I) and 68 kDa (type II) have been detected by protein analysis, the former mainly, but not exclusively, on T cells and fibroblasts and the latter on B cells. cDNAs of both receptors have been cloned and expressed (Solari, 1990; Ruhl *et al.*, 1992). High- and low-affinity binding sites for both IL-1α and IL-1β may be co-located on one or both receptors (Solari, 1990; Slack *et al.*, 1993). Both IL-1 receptors contain an immunoglobulin-like domain which binds IL-1, but the overall homology of this region in type I and type II receptors is only 28% (McMahan *et al.*, 1991; Ruhl *et al.*, 1992). It has been reported that signal transduction is exclusively mediated through receptor type I, which has an intracellular domain of 217 amino acids (Sims *et al.*, 1993). Despite lacking homology to any known protein, this intracellular domain is sufficiently long to encode for yet unidentified enzymatic function. In contrast, IL-1 receptor type II has a short intracellular domain of only 29 amino acids and might trap IL-1 without subsequent signal transduction, thus regulating IL-1-induced biological responses (McMahan *et al.*, 1991; Colotta *et al.*, 1993).

While IL-1 is a known growth factor for T and B cells, its potential significance for tumour cell proliferation is less clear. Some tumours appear to be stimulated, but growth of others is inhibited *in vitro* (Michiel and Oppenheim, 1992). Binding of tumour cells to endothelial cells *in vitro* and to blood vessel endothelium *in vivo* is enhanced under the influence of IL-1 (Giavazzi *et al.*, 1990; Lauri *et al.*, 1990). Thus, IL-1 may play a role in the formation of metastases *in vivo*. However, it is presently unclear how growth of tumour cells is modulated by IL-1 and which factors control the cellular response pattern to IL-1.

Correspondence: A-R Hanauske
Received 15 August 1994; revised 2 December 1994; accepted 5 December 1994

IL-1 is currently undergoing clinical investigation for use in cancer patients. It is therefore of interest to study the effects of IL-1 on tumour cell proliferation. The purpose of the present study was to evaluate the effects of IL-1α and IL-1β on freshly explanted human tumour cells and to reproduce the results using human renal carcinoma cell lines in order to scrutinise IL-1 receptor expression and IL-1-mediated cellular responses in human cancer cells.

### Materials and methods

### *Materials*

Human recombinant IL-1α was a generous gift from Hoffman LaRoche (Nutley, NJ, USA). [$^3$H]Thymidine, IL-1β and [$^{125}$I]IL-1α were purchased from Amersham-Buchler (Braunschweig, Germany). Antibodies against IL-1α and IL-1β and against IL-1 receptor types I and II were from Genzyme (Cambridge, MA, USA). Protein G Sepharose and disuccinimidylsuberate (DSS) were from Pierce (Rockford, IL, USA). Glassfibre filters were from Whatman (Clifton, NJ, USA). HEPES buffer solution as well as all tissue culture media, sera and additives were from Gibco-BRL (Eggenstein, Germany).

### *Cell lines and cell culture media*

Human renal cancer cell lines were purchased from the American Type Culture Collection (Rockville, MD, USA) and cultured in the following media:

A-498: modified Eagle's medium containing 10% fetal calf serum (FCS), non-essential amino acids and 1 mM sodium pyruvate;

Caki-1: McCoy's 5A medium containing 10% FCS;

Caki-2: McCoy's 5A medium containing 10% FCS;

ACHN: modified Eagle's medium containing 10% FCS and non-essential amino acids.

### *Soft-agar cloning*

Single-cell suspensions of solid human tumours were prepared mechanically; cultured cells were harvested with 0.25% trypsin/1 mM EDTA. Cells were seeded in 100 μl capillaries (six capillaries for each concentration) at a density of 40 000–62 000 cells per capillary in CMRL 1066 (cells from solid tumours) or 8000–20 000 cells $ml^{-1}$ in the tissue culture medium (cell lines) with 0.3% agar with or without increasing concentrations of IL-1α or IL-1β. Concentrations of FCS were as indicated in the text or legends to figures for different experiments. After incubation at 37°C for 3 weeks, colonies were examined and counted by light microscopy.

*Monolayer assay*

Approximately $2 \times 10^4$ cells were seeded in 24-well tissue culture plates in the tissue culture medium with concentrations of FCS as indicated with or without increasing concentrations of IL-1α or IL-1β. After various periods of incubation, cells were harvested with 0.25% trypsin/1 mM EDTA and counted.

*[$^3$H]Thymidine incorporation*

Cells were seeded in 96-well tissue culture plates ($1-5 \times 10^3$ cells per well) in medium with concentrations of FCS as indicated. After 3 days, the medium was changed and IL-1α or IL-1β was added. Incubation periods were 24, 48, 72, or 96 h. [$^3$H]Thymidine (0.75 μCi) was added 4 h before the end of the incubation period. Thereafter, cells were washed and harvested onto micro-glassfibre filters and the incorporated radioactivity was determined by β-scintillation counting.

*Enzyme-linked immunosorbent assay (ELISA)*

The conditioned media of the cell lines were studied for IL-1α and IL-1β using the Biotrak ELISA system (Amersham-Buchler, Braunschweig, Germany) as described by the manufacturer.

*IL-1 radioreceptor assay*

Cells were seeded in 24-well plates with or without IL-1α. When subconfluent, cells were washed once with binding medium pH 3.0 for 30 s in order to remove bound ligand and neutralised twice with binding medium (tissue culture medium with 20 mM HEPES, pH 7.0). [$^{125}$I]IL-1α was added at the indicated concentrations for direct binding assays and at a concentration of 80 pM and with increasing concentrations of unlabelled IL-1α for competition assays. Plates were incubated at 4°C for 3 h, then washed four times with phosphate-buffered saline (PBS) (140 mM sodium chloride, 30 mM potassium chloride, 6.5 mM disodium hydrogen phosphate, 1.2 mM potassium dihydrogen phosphate pH 7.2). Cells were lysed with 1 ml of 0.5% sodium dodecyl sulphate (SDS) and radioactivity was determined in a gamma-counter.

*Ligand–receptor cross-linking and immunoprecipitation*

Cells (confluency 90%) were incubated with 5 ng $ml^{-1}$ IL-1β for 3 h at 4°C and washed four times with ice-cold PBS. Cross-linking was performed with DSS (1 mg $ml^{-1}$) in PBS for 1 h at 4°C. Cells were washed twice with PBS, harvested wtih 20 mM EDTA in PBS and lysed with 1% Triton X-100 in PBS. Cell debris was removed by centrifugation. Lysates were diluted to a concentration of 0.5 mg $ml^{-1}$ protein with NET buffer (50 mM Tris–HCl pH 8.0, 150 mM sodium chloride, 0.1% Nonidet-P40, 1 mM EDTA, 0.25% gelatin) and incubated with antibodies against IL-1β at a concentration of 10 μg $ml^{-1}$ for 1 h at 4°C. Protein G–cellulose was added and the incubation was continued for 1 h at 4°C. Unbound proteins were removed by five washes with NET buffer. SDS–polyacrylamide gel loading buffer (25 mM Tris–HCl pH 6.8, 2% SDS, 1% 2-mercaptoethanol, 10% glycerin, 0.25% bromophenol blue) was added to the final protein G–cellulose pellet and samples were boiled for 5 min and cooled on ice. After electrophoresis on 10% acrylamide gels, proteins were blotted onto nitrocellulose filters (Laemmli, 1970). Filters were subsequently incubated with the antibody against IL-1β and a horseradish peroxidase-coupled anti-mouse antibody and stained with diaminobenzidine.

For direct immunoprecipitations of the receptor, cell lysates were incubated with antibodies against IL-1 receptor type I or II without prior cross-linking. In this case, Western blots were treated with anti-receptor antibodies.

### Results

Freshly explanted human tumour cells were cloned in soft agar with or without IL-1α or IL-1β (0.1–100 ng $ml^{-1}$) to investigate the influence of IL-1 on the clonogenic growth of human tumour cells (Buick and Salmon, 1980; Salmon and Salmon, 1980). Cell viability and cloning efficiency varied depending on the individual tumour examined. All results are expressed as colony survival relative to untreated controls. Endotoxin controls were included when preparations of IL-1-containing endotoxin were used. A tumour specimen was regarded as being stimulated if the number of colonies increased to more than 1.5 times control and as inhibited if the number of colonies decreased to less than 0.5 times control (Clark and Von Hoff, 1983).

Eighteen per cent of renal carcinomas were stimulated by IL-1α and thus were the most sensitive subgroup (Table I). Similar data were obtained with IL-1β (data not shown). The sensitivity of individual tumours was concentration dependent and reached 1.83 times control (Figure 1). Inhibition of clonogenic growth was observed in 4% of tumours without a clear relationship to the IL-1 concentration used.

Data obtained with freshly explanted human tumour cells were reproduced with established cell lines. Care was taken to keep the passage number as low as possible (between 15 and 50) and relatively constant for each cell line. Of the four cell lines used, two were from primary tumours (Caki-2 and A498), one was from a skin metastasis of a renal carcinoma (Caki-1) and one from a pleural effusion of a patient with widely metastatic renal carcinoma (ACHN). All cell lines were tested for endogenous production of IL-1α and IL-1β and were found not to release either cytokine into the medium (data not shown).

Clonogenic growth of tumour cell lines from primary cancers was stimulated in a concentration-dependent manner by IL-1α and IL-1β. With Caki-2 cells, stimulation was observed in tissue culture medium containing 10% FCS and reached 3.4 times control at 10 ng $ml^{-1}$ IL-1β and 2.5 times control at 100 ng IL-1α (Figure 2a). In contrast, A-498 cells were only stimulated after serum deprivation (1% FCS) with maximal effect of 2.0 times control at 100 ng $ml^{-1}$ IL-1α and 2.4 times control at 100 ng $ml^{-1}$ IL-1β (Figure 2b). Two cell

**Table I** Growth modulation of freshly explanted human tumours by increasing concentrations of interleukin 1α

| | | *0.1* | | *1.0* | | *10* | | *100* | |
|---|---|---|---|---|---|---|---|---|---|
| *Tumour type* | *No.* | *Stimulated* | *Inhibited* | *Stimulated* | *Inhibited* | *Stimulated* | *Inhibited* | *Stimulated* | *Inhibited* |
| Kidney | 22 | 3 | 0 | 2 | 2 | 4 | 1 | 3 | 1 |
| Colon | 9 | 0 | 0 | 0 | 0 | 0 | 1 | 0 | 0 |
| Stomach | 2 | 0 | 0 | 0 | 0 | 0 | 0 | 0 | 0 |
| Others | 11 | 2 | 0 | 2 | 0 | 2 | 0 | 4 | 0 |
| Total | 44 | 5 | 0 | 4 | 2 | 6 | 2 | 7 | 1 |

Specimens were considered to be stimulated if the average colony formation was ⩾1.5 times control and to be inhibited if the average colony formation was ⩽0.5 times control (Clark and Von Hoff, 1983).

**Figure 1** Representative concentration–response curves to IL-1α from two freshly explanted human renal carcinomas. Data represent mean and standard deviation of 4–6 assays.

lines from metastatic tumours (Caki-1, ACHN) failed to react to IL-1.

The rate of cell division of A-498 and Caki-2 cells in the absence or presence of IL-1α or IL-1β was determined by [$^3$H]thymidine incorporation and monolayer growth assays. Concentrations of IL-1α or IL-1β between 0.1 and 10 ng $ml^{-1}$ were tested using various concentrations of serum (0.1% BSA, 1% FCS, 10% FCS) and different incubation periods. IL-1α and IL-1β increased [$^3$H]thymidine incorporation into Caki-2 cells consistently and in a concentration-dependent manner. With 10% FCS and 10 ng $ml^{-1}$ IL-1β, stimulation was maximal after 48 h (1.4 times control, Figure 3a), whereas with 1% FCS maximal stimulation was reached only after 72 h or later (1.4 times control, data not shown). Also, monolayer growth was enhanced by IL-1α and IL-1β up to 1.5 times control in 10% FCS (Figure 3b). In contrast, IL-1 decreased [$^3$H]thymidine incorporation into A-498 cells (0.5 times control at 10 ng $ml^{-1}$ IL-1α or IL-1β, 1% FCS, Figure 3c). Similar results were obtained in a monolayer growth experiment. After 72 h of incubation, cell numbers were 0.5 times control after incubation with 0.1 or 10 ng $ml^{-1}$ IL-1 (Figure 3d). As in soft-agar cloning experiments, IL-1 modulation of A-498 cell growth was only observed under conditions of serum deprivation.

[$^{125}$I]IL-1 receptor assays were performed with all four cell lines to determine receptor number and binding affinity. [$^{125}$I]IL-1α did not bind to Caki-1 and ACHN cells (data not shown). A-498 cells were found to bind [$^{125}$I]IL-1α specifically and in a saturable manner. Scatchard analysis indicated that there are two classes of binding sites for IL-1α on these cells: high-affinity receptors ($K_D$ $4.5 \times 10^{-11}$ M, 150–250 receptors per cell) and low-affinity receptors ($K_D$ $1.3 \times 10^{-9}$ M, 2500 receptors per cell) (Figure 4). Interestingly, no specific binding to Caki-2 cells was detectable with this technique.

In order to evaluate whether IL-1 could modulate its own receptor, A-498 cells were incubated with increasing concentrations of IL-1α for 48 h. Receptor binding of [$^{125}$I]IL-1α was subsequently determined by incubation for 3 h at 4°C with 270 pM [$^{125}$I]IL-1α. Concentrations of more than 1 ng $ml^{-1}$ IL-1α were found to reduce [$^{125}$I]IL-1α binding by up to 50% (Figure 5a). The influence of IL-1α on [$^{125}$I]IL-1α binding was also time dependent: short periods (up to 30 min) of incubation with 2 ng $ml^{-1}$ IL-1α slightly enhanced IL-1α binding, whereas longer incubation periods reduced IL-1α binding to 40% of control values (Figure 5b). Preliminary data from Scatchard analyses of IL-1 radioreceptor assays with and without IL-1α preincubation indicate that reduced IL-1α binding after IL-1α preincubation is due to reduced affinity of the high-affinity receptors rather than to reduced receptor numbers (data not shown).

**Figure 2** (**a**) Stimulation of clonogenic growth of Caki-2 human renal carcinoma cells by IL-1α and IL-1β (10% FCS). (**b**) Stimulation of clonogenic growth of A-498 human renal carcinoma cells by IL-1α and IL-1β (1% and 10% FCS). Data represent mean and standard deviation of six assays.

We next attempted to characterise further IL-1 binding sites on Caki-2 and A-498 cells as either IL-1 receptor type I or type II. For this purpose, we performed soft-agar cloning experiments with Caki-2 cells in the presence of 2 ng $ml^{-1}$ IL-1β and increasing concentrations of antibodies against IL-1 receptor type I or type II. Antibodies alone were shown not to influence clonogenic growth of the cells. Only antibodies against IL-1 receptor type I inhibited IL-1-induced stimulation of clonogenic growth, whereas antibodies against IL-1 receptor type II had no effect on clonogenic proliferation (Figure 6). Signal transduction of IL-1 and Caki-2 cells therefore appears to occur through receptor type I. Similar results were obtained with A-498 cells (data not shown).

Next, we studied whether IL-1 receptor type I is the only form of receptor species expressed by renal carcinoma cell lines. Using immunoprecipitation techniques, we identified a band of approximately 100 kDa in a subsequent Western blot in A-498 cells, in which IL-1 receptor type I (80 kDa) is cross-linked to IL-1 (17 kDa) (Figure 7). To confirm the identity of the receptor as type I we performed immunoprecipitations using anti-IL-1 receptor type I or type II antibodies directly to precipitate the receptor from fresh cell lysates without prior cross-linking. In A-498 cells a signal was observed using anti-IL-1 receptor type I antibodies. No signal was detected using anti-IL-1 receptor type II antibodies (data not shown). This indicates that binding of IL-1 to A-498 cells occurs via receptor type I. No receptors were identified on Caki-2 cells, probably because the sen-

**Figure 3** (**a**) [$^3$H]Thymidine incorporation into Caki-2 cells (10% FCS) under the influence of IL-1$\beta$. (**b**) Monolayer cell growth of Caki-2 cells (10% FCS) under the influence of IL-1$\alpha$ and IL-1$\beta$ (10 ng ml$^{-1}$). (**c**) [$^3$H]Thymidine incorporation into A-498 cells (1% FCS) under the influence of IL-1$\beta$. (**d**) Monolayer cell growth of A-498 cells (1% FCS) under the influence of IL-1$\alpha$ and IL-1$\beta$ (10 ng ml$^{-1}$).

**Figure 4** Scatchard analysis of [$^{125}$I]IL-1$\alpha$ receptor binding to A-498 renal cancer cells. Inserts show binding curves. (**a**) Direct binding studies. (**b**) Competitive binding studies.

**Figure 5** (**a**) Ligand-induced down-regulation of [$^{125}$I]IL-1α binding to A-498 cells after preincubation with IL-1α for 48 h. (**b**) Influence of preincubation time of IL-1α (2 ng ml$^{-1}$) on [$^{125}$I]IL-1α binding.

sitivity of the available methods is insufficient to identify these weakly expressed molecules.

### Discussion

Though primarily identified as a haematopoietic growth factor, IL-1 now has to be regarded as a peptide with growth-modulating effects on many cell types, including human tumour cells. At present, it is not known whether growth stimulation or inhibition of tumour cells influences tumour biology *in vivo*. However, the beneficial effects of IL-1 on haematopoiesis may be used to decrease myelotoxic effects induced by cytotoxic chemotherapy.

*In vitro*, cloning of freshly explanted human tumour cells has demonstrated that exposure to IL-1 may modulate clonogenic growth in a subset of tumours. In confirmation of earlier results from our group, most stimulated tumours were renal carcinomas (Donné *et al.*, 1992). Since it is largely accepted that tumour cell lines are a good *in vitro* model for mechanistic investigations, renal carcinoma cell lines were used for further studies, bearing in mind that some changes may have occurred during *in vitro* passaging. In agreement with our results from clinical tumour specimens, the clonogenicity of two out of four human renal carcinoma cell lines increased under the influence of IL-1. Interestingly, both responsive cell lines (Caki-2 and A-498) had been established from primary tumours, while two cell lines from metastatic

**Figure 6** Inhibition of IL-1α-induced stimulation of clonogenic growth of Caki-2 cells by monoclonal antibodies to IL-1 receptor type I and II. Data represent mean and standard deviation of six assays.

**Figure 7** Immunoprecipitation of IL-1 receptor type I after cross-linking to IL-1α, detected by a monoclonal antibody to IL-1α. Size markers are given in kDa. Lane A, Caki-2 cells; lane B, A-498 cells. Arrows indicate immunoprecipitated IL-1 receptor type I cross-linked to IL-1α (100 kDa), IgG1 antibody to IL-1α (48 kDa) and IL-1α (17 kDa).

lesions lacked IL-1 sensitivity in clonogenic growth experiments. For Caki-2, cell proliferation as determined by monolayer growth and [$^3$H]thymidine uptake was also enhanced. In contrast, A-498 cells showed a decrease in overall [$^3$H]thymidine incorporation and monolayer proliferation. This indicates that IL-1 may differentially act on cells that retain clonogenic capabilities.

In a next step we found A-498 cells to bind IL-1 in a saturable manner. Scatchard analysis of the binding data revealed high-affinity receptors with low density and low-affinity binding sites with higher density. The number of IL-1 receptors per cell and their dissociation constants are in the range of those published for other human tumour cell lines, although other investigators only reported on a single class of receptors. Breast cancer cells (MCF-7) express 2500 IL-1 receptors per cell with a $K_D$ of 200 pM (Paciotti and Tamarkin, 1988). The human ovarian carcinoma cell line Ovcar-3 has been reported to express 7800 sites per cell with a $K_D$ of 55 pM (Tsai and Gaffney, 1987). Lower receptor numbers have been found on NIM-1 human thyroid carcinoma cells (664 molecules per cell, $K_D$ 110 pM) as well as in MDA-MB-415 breast cancer cells (700 per cell, $K_D$ 880 pM) (Gaffney *et al.*, 1988; Zeki *et al.*, 1993). Although Caki-2 cells were more sensitive to the proliferative effects of IL-1, cell-surface receptors could not be identified using [$^{125}$I]IL-1. The reasons for this are unclear but may be related to low receptor numbers on these cells and sensitivity limits of the assays used.

In order to characterise further the functional status of IL-1 receptors on A-498 cells, we performed binding experiments after preincubation with unlabelled ligand. We found that IL-1α reduced binding of [$^{125}$I]IL-1α to its receptor. The concentration of unlabelled ligand was saturating at least for the high-affinity binding sites. These data are in accordance with published reports that saturating concentrations of IL-1α or IL-1β down-regulate cell-surface expression of IL-1 receptor by destabilising its mRNA (Ye *et al.*, 1992). However, Scatchard analyses clearly indicate that reduced binding to A-498 cells is not due to reduced receptor number, but rather to reduced receptor affinity. In this context it may be interesting to note that receptor number might have little or no influence on the extent of the biological response, since a receptor occupancy of 1–100 molecules per cell has been reported to be sufficient for efficient signal transduction (Dower *et al.*, 1985). However, reduced affinity of high-affinity receptors may well weaken the biological signal, particularly at low concentrations of ligand.

While 250 high-affinity receptors on A-498 cells are sufficient for effective signal transduction, a far lower receptor number, which is too low for identification by radioreceptor assays, may account for IL-1-induced growth stimulation of Caki-2 cells.

From our experiments, we cannot exclude the possibility that Caki-1 and ACHN cells also express a small number of cell-surface receptors. However, if this is the case, binding of IL-1 to its receptor does not change the growth behaviour of these cells.

Early reports have postulated that both type I and type II IL-1 receptors are functional in ligand binding and capable of signal transduction. However, recent reports support the notion that only receptor type I can mediate IL-1-induced biological effects, at least in lymphocytes (Sims *et al.*, 1993). While the intracellular domain of this molecule with 217 amino acids is large enough for a yet unidentified enzymatic function, the intracellular domain of receptor type II is too short. Binding of IL-1 to receptor type II may reduce the effective IL-1 concentration and thus may have a regulatory function with regard to biological effects (Colotta *et al.*, 1993). In Caki-2 and A-498 cells, IL-1-induced stimulation of clonogenic growth was prevented by antibodies to receptor type I but not to receptor type II. This indicates that IL-1 signal transduction in these cells is also conferred via this receptor type. Similarly, binding of IL-1 to A-498 cells was inhibited by 50% by antibodies to receptor type I at the concentrations of antibody used. We therefore conclude that renal cancer cells mainly express IL-1 receptor type I. Inhibition of IL-1 binding by 25% by antibody to receptor type II may be explained by a lack of specificity of the antibodies used.

In confirmation of cell biological data, a single band of about 100 kDa was immunoprecipitated by antibodies to IL-1α after cross-linking of IL-1α to A-498 cells. The size of this band corresponds to IL-1 receptor type I (80 kDa) cross-linked to IL-1 (17 kDa). No band of minor size which might represent IL-1 receptor type II (60 kDa) was identified. We therefore hypothesise that binding of IL-1 to renal carcinoma cells occurs via IL-1 receptor type I and that this receptor is also responsible for signal transduction. Similar results have been obtained with MCF-7 cells, indicating a general role for receptor type I in IL-1 signal transduction not only in lymphocytes but also in cancer cells (Paciotti and Tamarkin, 1988).

In summary, our data indicate that IL-1 can modulate growth of human renal cancer cells and that this effect is conferred by IL-1 receptor type I.

**Abbreviations** DSS, disuccinimidylsuberate; HEPES, 4-(2-hydroxyethyl)-1-piperazineethanesulphonic acid; SDS, sodium docecyl sulphate; FCS, fetal calf serum; BSA, bovine serum albumin; ELISA, enzyme-linked immunosorbent assay.

**Acknowledgements**
Supported by grant 90.005.1 by the Wilhelm Sander Stiftung.

### References

BUICK RN AND SALMON SE. (1980). In vitro clonogenicity of primary human tumor cells: quantitation and relationship to tumor stem cells. In *Progress in Clinical and Biological Research: Cloning of Human Tumor Stem Cells*, Vol. 48, pp. 15–23. Alan R. Liss: New York.

CLARK GM AND VON HOFF DD. (1983). Statistical considerations for in vitro/in vivo correlations using a cloning system. In *Human Tumor Drug Sensitivity Testing In Vitro*, Dendy P, Hill BT. (eds) pp. 225–233. Academic Press: New York.

COLOTTA F, RE F, MUZIO M & 7 others. (1993). Interleukin-1 type II receptor: a decoy target for IL-1 that is regulated by IL-4. *Science*, **261**, 472–475.

DONNE S, DE RIESE W, RAAB H-R & 7 others. (1992). Growth-modulating effects of interleukin-1α, interleukin-1β, and macrophage colony-stimulating factor in clonogenic tumor cells in vitro. In *Cytokines in Hemopoiesis, Oncology, and Aids II*, Freund M, Link H, Schmidt RE, Welte K. (eds) pp. 127–134. Springer: Berlin.

DOWER SK, KRONHEIM SR, MARCH CJ & 4 others. (1985). Detection and characterization of high affinity plasma membrane receptors for human interleukin-1. *J. Exp. Med.*, **162**, 501–515.

GAFFNEY EV, KOCH G, TSAI S, LOUCKS T AND LINGENFELTER SE. (1988). Correlation between human cell growth response to interleukin-1 and receptor binding. *Cancer Res.*, **48**, 5455–5459.

GIAVAZZI R, GAROFALO A, BANI MR & others. (1990). Interleukin 1-induced augmentation of experimental metastases from a human melanoma in nude mice. *Cancer Res.*, **50**, 4771–4775.

LAEMMLI UK. (1970). Cleavage of structural proteins during the assembly of the head of bacteriophage T4. *Nature*, **227**, 680.

LAURI D, BERTAMEU M, ORR FW, BASTIDA E, SAUDER D AND BUCHANAN MR. (1990). Interleukin-1 increases tumor cell adhesion to endothelial cells through an RGD dependent mechanism: in vitro and in vivo studies. *Clin. Exp. Metastasis*, **8**, 27–32.

MCMAHAN CJ, SLACK JL, MOSLEY B & 14 others. (1991). A novel IL-1 receptor, cloned from B cells by mammalian expression, is expressed in many cell types. *EMBO J.*, **10**, 2821–2832.

MICHIEL DF AND OPPENHEIM JJ. (1992). Cytokines as positive and negative regulators of tumor promotion and progression. *Cancer Biol.*, **3**, 3–15.

MIZEL SB, KILIAN PL, LEWIS, JC, PAGANELLI KA AND CHIZZONITE RA. (1987). The interleukin 1 receptor. Dynamics of interleukin 1 binding and internalization in T cells and fibroblasts. *J. Immunol.*, **138**, 2906–2912.

PACIOTTI GF AND TAMARKIN L. (1988). Interleukin-1 directly regulates hormone-dependent human breast cancer cell proliferation in vitro. *Mol. Endocrinol.*, **2**, 459–464.

RUHL S, SCHWABE M AND PLUZNIK DV. (1992). Interleukin-1 augments the expression of the interleukin-2 receptor α-chain in interleukin-6 stimulated myeloid cells by a transcriptional and posttranscriptional mechanism. *Exp. Hematol.*, **20**, 1208–1215.

SALMON ES AND SALMON SE. (1980). Morphologic studies of tumor colonies. In *Progress in Clinical and Biological Research: Cloning of Human Tumor Stem Cells*, Vol. 48, pp. 135–152. Alan R. Liss: New York.

SIMS JE, GAYLE MA, SLACK JL & 9 others. (1993). Interleukin-1 signaling occurs exclusively via the type I receptor. *Proc. Natl Acad. Sci. USA*, **90**, 6155–6159.

SLACK J, MCMAHAN CJ, WAUGH S & 4 others. (1993). Independent binding of interleukin-1α and interleukin-1β to type I and type II interleukin-1 receptors. *J. Biol. Chem.*, **268**, 2513–2524.

SOLARI R. (1990). Identification and distribution of two forms of the interleukin-1 receptor. *Cytokine*, **2**, 21–28.

TSAI SJ AND GAFFNEY EV. (1987). Modulation of cell proliferation by human recombinant interleukin-1 and immune interferon. *J. Natl Cancer Inst.*, **79**, 77–81.

YE K, KOCH K-C, CLARK BC AND DINARELLO CA. (1992). Interleukin-1 down-regulates gene and surface expression of interleukin-1 receptor type I by destabilizing its mRNA whereas interleukin-2 increases its expression. *Immunology*, **75**, 427–434.

ZEKI K, NAKANO Y, INOKUCHI N & 4 others. (1993). Autocrine stimulation of interleukin-1 in the growth of human thyroid carcinoma cell line NIM 1. *J. Clin. Endocrinol. Metab.*, **76**, 127–133.